# ﻿Taxonomic and nomenclatural notes on Chinese species of *Sarcophaga* Meigen, 1824 (Diptera, Sarcophagidae)

**DOI:** 10.3897/zookeys.1108.83267

**Published:** 2022-06-24

**Authors:** Chao Wang, Haoran Sun, Weibing Zhu, Thomas Pape, Qiyong Liu, Dong Zhang

**Affiliations:** 1 State Key Laboratory of Infectious Disease Prevention and Control, WHO Collaborating Centre for Vector Surveillance and Management, National Institute for Communicable Disease Control and Prevention, Chinese Center for Disease Control and Prevention, Beijing 102206, China Beijing Forestry University Beijing China; 2 School of Ecology and Nature Conservation, Beijing Forestry University, Beijing 100083, China National Institute for Communicable Disease Control and Prevention, Chinese Center for Disease Control and Prevention Beijing China; 3 Center for Excellence in Molecular Plant Science, Chinese Academy of Sciences, Shanghai 200032, China Center for Excellence in Molecular Plant Science, Chinese Academy of Sciences Shanghai China; 4 Natural History Museum of Denmark, University of Copenhagen, Universitetsparken 15, DK-2100, Copenhagen, Denmark University of Copenhagen Copenhagen Denmark

**Keywords:** *Leigongshanophaga*, *Magnicauda*, new synonyms, nomenclature, original spellings, revision, *Sarcophaga*, taxonomy

## Abstract

New taxonomic and nomenclatural data are provided for Chinese species of *Sarcophaga* Meigen, 1824. Eight new synonyms are proposed: two at the genus level, *Magnicauda* Wei, 2005 **syn. nov.** = *Sarcophaga* Meigen, 1824 and *Leigongshanophaga* Lehrer & Wei, 2010 **syn. nov.** = Sarcophaga Meigen, 1824, two at the subgenus level, *Magnicauda* Wei, 2005 **syn. nov.** = *Pterosarcophaga* Ye, 1981 and *Leigongshanophaga* Lehrer & Wei, 2010 **syn. nov.** = *Cornexcisia* Fan & Kano, 2000, and four at the species level, *Sarcophagacatoptosa* Wei & Yang, 2007 **syn. nov.** = *Sarcophagasuthep* Pape & Bänziger, 2003, *Pierretiadaozhenensis* Wei, 2005 **syn. nov.** = *Sarcophagasichotealini* (Rohdendorf, 1938), *Pierretiaautochthona* Wei & Yang, 2007 **syn. nov.** = Sarcophaga (Liosarcophaga) kanoi Park, 1962, and *Parasarcophagasimultaneousa* Wei & Yang, 2007 **syn. nov.** = *Sarcophagahuangshanensis* (Fan, 1964). Sarcophaga (Liosarcophaga) aegyptica Salem, 1935 is considered a senior synonym of Sarcophaga (Liosarcophaga) parkeri (Rohdendorf, 1937). Correct original spellings are established, by First Reviser action, for the genus-group names *Magnicauda* Wei, 2005 and *Pterosarcophaga* Ye, 1981 and for the species-group name *Magnicaudalinjiangensis* Wei, 2005. Chinese material of Sarcophaga (Bellieriomima) genuforceps, S. (Robineauella) huangshanensis (holotype and paratype), S. (Liosarcophaga) kanoi, and S. (L.) aegyptica is photographed for the first time.

## ﻿Introduction

*Sarcophaga* Meigen, 1824 (*sensu lato*) is by far the largest genus in the Sarcophagidae, and with upwards of a thousand species it is also one of the largest genera of Diptera (Whitmore et al. 2013; Wang et al. 2019, 2020; Evenhuis and Pape 2021). The genus is widespread, and the adults are very homogeneous in their external morphology and often recognizable at the species level only through a detailed study of the male terminalia (Buenaventura et al. 2017), for which professional skills as well as considerable experience are needed. The uniformity in external appearance stands in strong contrast to the marked structural complexity of the male terminalia, where phallic morphology in particular has diversified through the evolution of variously shaped appendages, the homologies of which are often obscure. The diversity and variability of the male terminalia, combined with the practical need to break up the large *Sarcophaga* (*sensu lato*) into smaller taxa, has brought about a high number of genus-level and species-level synonyms (Pape 1996; Wang et al. 2019, 2020). Ongoing studies of the Chinese fauna of *Sarcophaga* has led to the recognition of several new synonyms, which are presented here together with relevant taxonomic and nomenclatural details.

## ﻿Material and methods

Specimens examined or otherwise mentioned are deposited at the following institutions:

**CDCP** Center for Disease Control and Prevention of Anshun city, Guizhou province, China;

**MNHN**Muséum national d’Histoire naturelle, Paris, France;

**MBFU**Museum of Beijing Forestry University, Beijing, China;

**NHMD** Natural History Museum of Denmark;

**SECA**Shanghai Entomological Museum, Chinese Academy of Sciences, Shanghai, China;

**SMNH**Swedish Museum of Natural History.

Identifications were aided by the keys in the publication of Fan (1992), combined with extensive comparisons against specimens in the reference collections of MBFU and NHMD, supplemented by a library of images of male terminalia and the original descriptions. We follow Roback (1954), Downes (1965), Pape (1996), Pape and Dahlem (2010), Giroux et al. (2010), Richet et al. (2011), Whitmore et al. (2013), Buenaventura et al. (2017), and Buenaventura and Pape (2018) in a broad definition of the genus *Sarcophaga*. External morphology was examined with an Olympus SZX16 stereomicroscope, and photographs were taken with a Canon 600D camera mounted on the same microscope. Images were processed in Adobe Photoshop CS 6 (Adobe Systems, Inc., San Jose, CA, USA) and stacked in Helicon Focus 3.2 (Helicon Soft Ltd, Kharkov, Ukraine). Inked illustrations were done by tracing over a photograph or figures from the original descriptions. The International Code of Zoological Nomenclature (ICZN 1999) is referred to as “the Code”.

## ﻿Taxonomy and nomenclature

### ﻿Genus Sarcophaga

Taxon classificationAnimaliaDipteraSarcophagidae

Meigen, 1824

A6689F66-B1FE-55D1-87E6-DCB4FF82E22A

Sarcophaga Meigen, 1824: 305. Type species: Muscacarnaria Linnaeus, 1758, by subsequent designation of Partington (1837: 607).
Magnicauda Wei, 2005: 405. Type species: Magnicaudalinjiangensis Wei, 2005, by original designation. Syn. nov.
Maginicauda : Wei (2005: 409). Incorrect original spelling of Magnicauda, by First Reviser action in the present paper.
Leigongshanophaga Lehrer & Wei, 2010: 8. Type species: Sarcophagacatoptosa Wei & Yang, 2007 [= Sarcophagasuthep Pape & Bänziger, 2003], by original designation. Syn. nov. For other synonyms, see Pape (1996).

#### Remarks.

Verves and Khrokalo (2020: 204) proposed *Leigongshanophaga* Lehrer & Wei, 2010 as a new synonym of the valid genus *Rosellea* Rohdendorf, 1937, but Xue et al. (2011: 320) proposed the same earlier. As argued below, we consider *Sarcophagacatoptosa* Wei & Yang, 2007, which is the type species of *Leigongshanophaga* Lehrer & Wei, 2010, to be a synonym of *Sarcophagasuthep* Pape & Bänziger, 2003, syn. nov., and we follow Wang et al. (2019) in treating this species in SarcophagasubgenusCornexcisia Fan & Kano, 2000.

### Subgenus ﻿ Bellieriomima

Taxon classificationAnimaliaDipteraSarcophagidae

Rohdendorf, 1937

CBAC100A-5268-5891-810F-9F7340DF7F58

Bellieriomima Rohdendorf, 1937: 164 (as subgenus of Thyrsocnema Enderlein, 1928). Type species: Sarcophagalaciniata Pandellé, 1896 [= Sarcophagasubulata Pandellé, 1896], by original designation.

### ﻿ Sarcophaga (Bellieriomima) genuforceps

Taxon classificationAnimaliaDipteraSarcophagidae

Thomas, 1949

9114D38D-19F2-5B0C-81C6-2AF808EA192A

Figs 1 , 2

Sarcophaga genuforceps Thomas, 1949: 172. China, Sichuan, Chungking, Chinyunshan.
Pierretia catharosa Wei & Yang, 2007: 530. China, Guizhou, Leigongshan.

#### Material examined.

1♂, China, Zhejiang, Tianmu Mountain, 600–1100 m, 30.vi.1964, Huitai Fang leg. (SECA).

**Figure 1.**
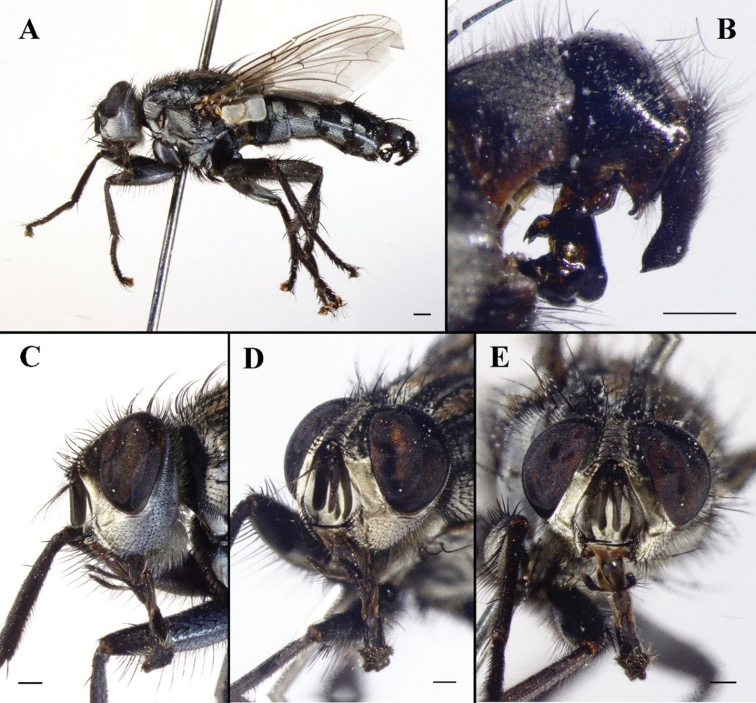
Sarcophaga (Bellieriomima) genuforceps Thomas, 1949; male (China, Zhejiang, Tianmu Mountain; in SECA) **A** habitus, lateral view **B** terminalia, lateral view **C** head, lateral view **D** head, anterolateral view **E** head, anterior view. Scale bars: 1 mm.

#### Remarks.

**Figure 2.**
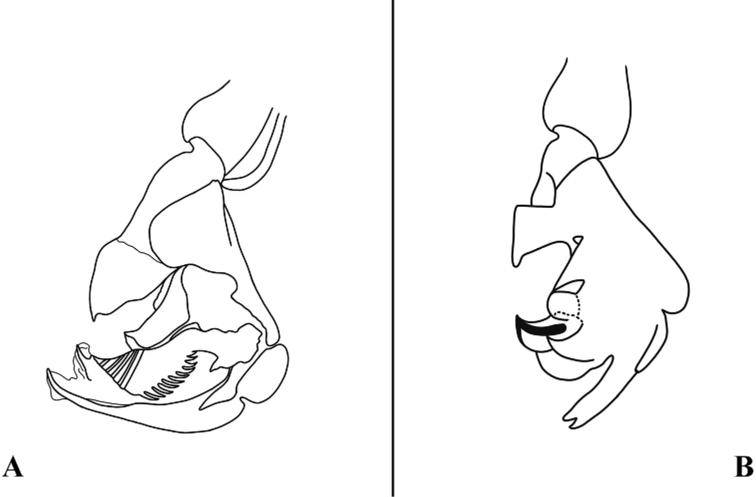
Sarcophaga (Bellieriomima) genuforceps Thomas, 1949; phallus, lateral view **A** adapted from Lehrer (2012)**B** adapted from Wei and Yang (2007, as *Pierretiacatharosa*).

The holotype of *Pierretiacatharosa* is deposited in CDCP and not currently available for loan and study. Verves (2020: 36) listed *P.catharosa* as a junior synonym of *S.genuforceps*, although not as a new synonym. Wei and Yang (2007) gave a detailed description and a somewhat schematical illustration of the phallus (Fig. 2B), which is here considered sufficient justification for the synonymy. Xue and Verves (2009: 53) considered *S.genuforceps* to belong to Pachystyleta Fan & Chen, 1992, as a subgenus of Myorhina Robineau-Desvoidy, 1830, whereas Lehrer (2010: 18) raised *Pachystyleta* to genus rank. We prefer to follow the classification of Pape (1996), with *Pachystyleta* as a synonym of *Bellieriomima* and the latter as a subgenus of *Sarcophaga* (*sensu lato*).

### Subgenus ﻿ Cornexcisia

Taxon classificationAnimaliaDipteraSarcophagidae

Fan & Kano, 2000

0980FF0A-4D4F-5640-9AB7-02B15BCDCD3E

Cornexcisia Fan & Kano, 2000: 251. Type species: Cornexcisialongicornuta Fan & Kano, 2000, by original designation.
Leigongshanophaga Lehrer & Wei, 2010: 8. Type species: Sarcophagacatoptosa Wei & Yang, 2007 [= Sarcophagasuthep Pape & Bänziger, 2003], by original designation. Syn. nov.

### ﻿ Sarcophaga (Cornexcisia) suthep

Taxon classificationAnimaliaDipteraSarcophagidae

Pape & Bänziger, 2003

3A733BD9-537D-5D94-9F11-822EB06BDDC0

Fig. 3

Sarcophaga suthep Pape & Bänziger, 2003: 52. Thailand, Chiang Mai Province, Doi Suthep.
Sarcophaga catoptosa Wei & Yang, 2007: 531. China, Guizhou, Leigongshan. Syn. nov.
Sarcophaga sutheb : Wei and Yang 2007: 532. Incorrect subsequent spelling of S.suthep Pape & Bänziger, 2003.

#### Material examined.

***Holotype*** of *S.suthep*: ♂, Thailand, Chiang Mai Province, Doi Suthep, above Sangwal School, 1240 m, 28.viii.2000, H. Bänziger (in SMNH; specimen dissected and with terminalia glued to a piece of cardboard pinned below the specimen).

#### Remarks.

**Figure 3.**
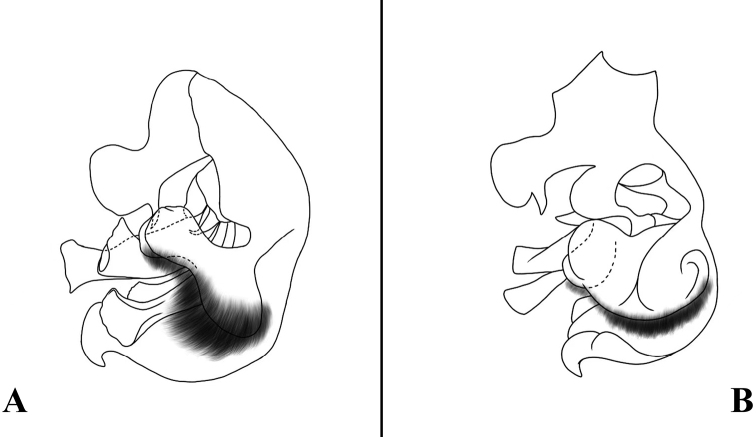
Sarcophaga (Cornexcisia) suthep Pape & Bänziger, 2003; phallus, lateral view **A** adapted from Pape and Bänziger (2003)**B** adapted from Wei and Yang (2007, as *Sarcophagacatoptosa*).

The holotype of *Sarcophagacatoptosa* is deposited in CDCP and not currently available for loan and study. Wei and Yang (2007) described the lateral styli as bifurcated at the base and expanded at the apex (Fig. 3). This unique character in *Sarcophaga* is shared by *S.suthep* and other species assigned to the subgenus Cornexcisia. We consider the following compelling similarities between the nominal species *S.suthep* and *S.catoptosa*, as assessed from the illustrations of the phallus (Fig. 3), to justify the proposed synonymy: vesica of identical shape; juxta, harpes and lateral styli differing only by small differences in the outline, and this involves membranous parts that are often presenting themselves very differently due to shrinking during drying or other preparation. Wei and Yang (2007) stressed the following difference between *catoptosa* and *suthep*: the protuberance of former cerci is slightly narrower than the latter in dorsal view and the hind margin of former pregonite is wavy bending with a sharper tip, but those differences are minor. They still have the same shape, only varying in degree. Therefore, we consider these to be intraspecific differences.

### Subgenus ﻿ Liosarcophaga

Taxon classificationAnimaliaDipteraSarcophagidae

Enderlein, 1928

0F9AA0F0-2370-546D-824B-78E163BD2067

Liosarcophaga Enderlein, 1928:18. Type species: Cynomyamadeirensis Schiner, 1868, by original designation.

### ﻿ Sarcophaga (Liosarcophaga) aegyptica

Taxon classificationAnimaliaDipteraSarcophagidae

Salem, 1935

19DD21C3-9F9E-5B86-B486-AFA7ECEBCC4C

Fig. 4

Sarcophaga dux aegyptica Salem, 1935: 56. Egypt, Alexandria; Egypt, Abbassieh; Egypt, Monsouriah.
Parasarcophaga (Liosarcophaga) parkeri Rohdendorf, 1937: 217. Ukraine, south shore of Crimea.

#### Material examined.

1♂, China, Qinghai, Minhe, 22.vii.1976, Shaoyuan Ma leg. (SECA).

#### Remarks.

There has been disagreement among authors as to whether *Parasarcophagaparkeri* is a valid species or a junior synonym of *S.aegyptica*. Rohdendorf (1937) evidently knew Salem’s (1935) work on *Sarcophaga* (s.l.) from Egypt, but he did not study any material identified as *S.aegyptica* and therefore quoted Salem’s description. Furthermore, the diagnostic differences in the shape of the juxtal arms and harpes outlined in the key by Rohdendorf (1937: 440) were assessed based on Salem’s illustrations. Gregor and Povolný (1960) synonymized the two nominal species, which was accepted by Rohdendorf (1970), and these taxa have since been considered either as separate species, e.g., by Lehrer (1995), Pape (1996), El-Ahmady et al. (2018), and Verves and Khrokalo (2020), or as synonyms, e.g., by Xue and Chao (1998), Nandi (2002), Povolný and Hula (2004), and Richet et al. (2011). The recent conspectus of Egyptian species of *Sarcophaga* (s.l.) by El-Ahmady et al. (2018) separated *aegyptica* and *parkeri* by vesica with two short processes apically and narrow harpes (*aegyptica*) versus vesica with three short processes apically and broad harpes (*parkeri*). The material at our disposal was not sufficient for a thorough assessment of the relevant morphological characters, but we have the impression that both the vesica and the harpes are variable structures, which furthermore present themselves very differently depending on the type of preparation and condition of the specimen. We have therefore chosen a conservative approach and consider the two nominal taxa as synonyms.

**Figure 4.**
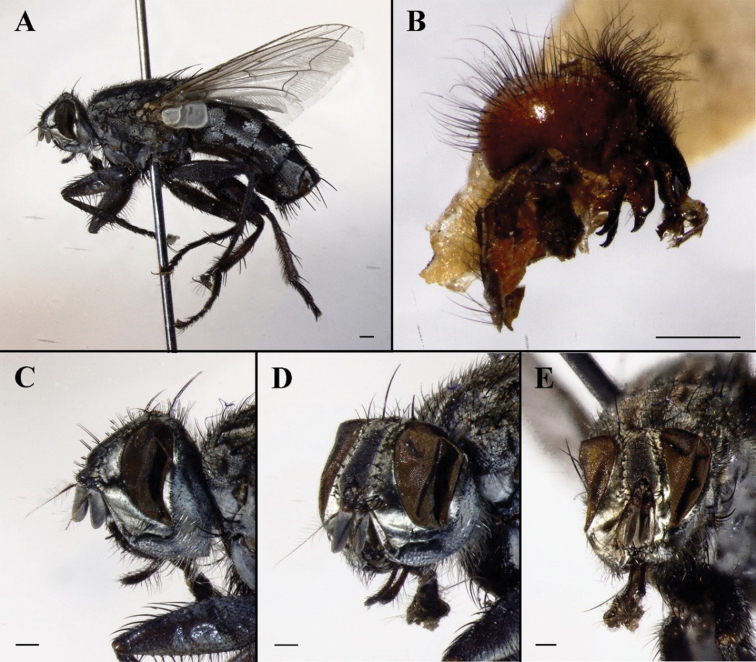
Sarcophaga (Liosarcophaga) aegyptica Salem, 1935; male (China, Qinghai; in SECA) **A** habitus, lateral view **B** terminalia, lateral view **C** head, lateral view **D** head, anterolateral view **E** head, anterior view. Scale bars: 1 mm.

### ﻿ Sarcophaga (Liosarcophaga) kanoi

Taxon classificationAnimaliaDipteraSarcophagidae

Park, 1962

DE88A269-0585-57AC-B3D1-5A3CE79180C9

Fig. 5

Sarcophaga (Liosarcophaga) kanoi Park, 1962: 6. South Korea, Taegu, Mt Pal-gong.
Pierretia autochthona Wei & Yang, 2007: 529. China, Guizhou, Leigongshan. Syn. nov.
Pierretia autochtona : Verves 2020: 37, incorrect subsequent spelling of P.autochthona.

#### Material examined.

1♂, China, Shanghai (Zi-Ka-Wei), 3.ix.1917, no further data (MNHN). 1♂, China, Hunan, Anxiang, Guandang, 20–21.vii.2012, Ming Zhang leg.; 1♂, China, Hunan, Anxiang, Guandang, 7.vii.2013, Ming Zhang leg.; 3♂♂, China, Hubei, Shishou, Gaoling, 8.vii.2013, Ming Zhang leg.; 1♂, China, Beijing, Beijing Forestry University, 9.vii.2016, Miao Jiang & Yunyun Gao leg. (MBFU).

**Figure 5.**
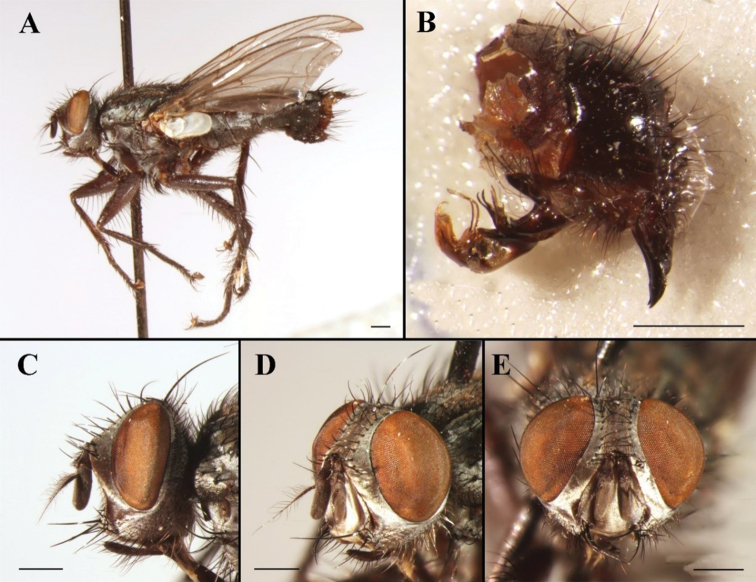
Sarcophaga (Liosarcophaga) kanoi Park, 1962; male (China, Hubei; in MBFU) **A** habitus, lateral view **B** terminalia, lateral view **C** head, lateral view **D** head, anterolateral view **E** head, anterior view. Scale bars: 1 mm.

#### Remarks.

**Figure 6.**
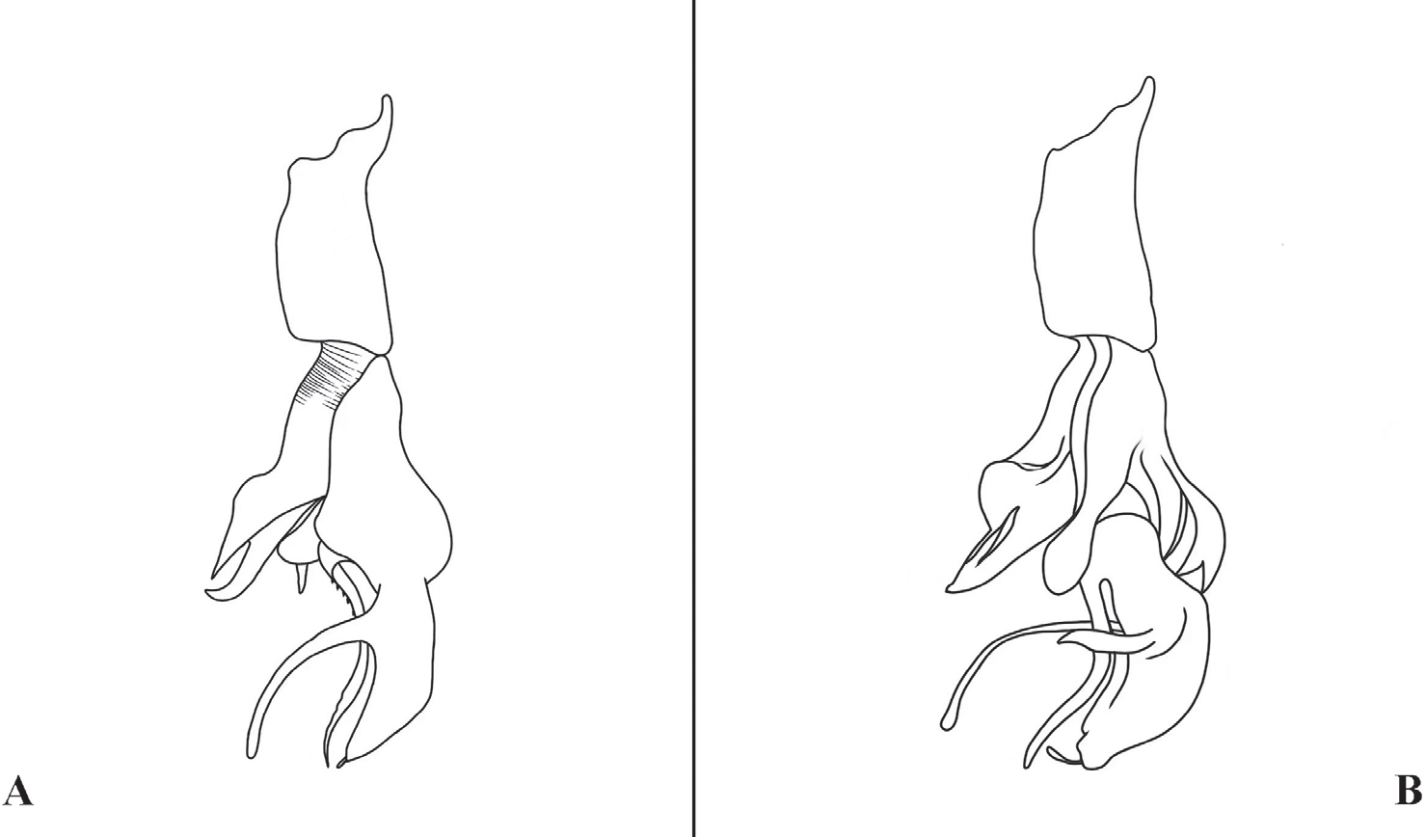
Sarcophaga (Liosarcophaga) kanoi Park, 1962; phallus, lateral view **A** adapted from Lehrer (2012)**B** adapted from Wei & Yang (2007, as *Pierretiaautochthona*).

Wei and Yang (2007) considered *P.autochthona* as close to S. (Pseudothyrsocnema) caudagalli Böttcher, 1912, but we are here proposing a synonymy with S. (L.) kanoi. Wei and Yang (2007: fig. 72) illustrated the phallus of the holotype of *P.autochthona* as having a short, arm-like extension arising from the left lateral part of the distiphallus (probably the proximal part of the juxta) and a long, slender, process arising from the right lateral part of the distiphallus (Fig. 6). We consider this apparent asymmetry to be an artefact, and possibly an inaccuracy of the original illustration. This could not be confirmed because the holotype of *P.autochthona*, deposited in CDCP, has not been available for study through ordinary loan.

### ﻿Subgenus Phallantha

Taxon classificationAnimaliaDipteraSarcophagidae

Rohdendorf, 1938

38A8ACF1-0820-512D-A425-B22D284C1CE5

Phallantha Rohdendorf, 1938: 101. Type species: Phallanthasichotealini Rohdendorf, 1938, by original designation.

### ﻿ Sarcophaga (Phallantha) sichotealini

Taxon classificationAnimaliaDipteraSarcophagidae

(Rohdendorf, 1938)

DF55E647-EF84-568E-884D-6F20AF2E9B92

Fig. 7

Phallantha sichotealini Rohdendorf, 1938: 102. Russia, Primorye, Sikhote-Alin State Reservation.
Pierretia daozhenensis Wei in Wei & Yang, 2005: 424. China, Guizhou, Daozhen, Dashahe. Syn. nov.

#### Material examined.

1♂, Russia, Primorye, SE Ussurijsk, 8.viii.1983, A. Ozerov leg. (NHMD). 1♂, China, Sichuan, Baoxing, 8.v.1981, unknown leg.; 1♂, China, Sichuan, Ya’an, 29.iv.2002, unknown leg. (SECA).

**Figure 7.**
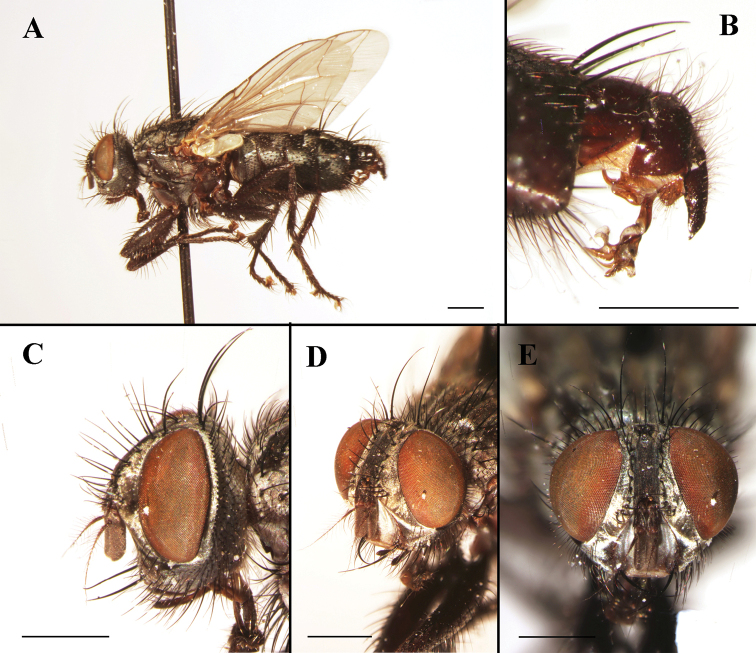
Sarcophaga (Phallantha) sichotealini (Rohdendorf, 1938); male (China, Sichuan; in SECA) **A** habitus, lateral view **B** terminalia, lateral view **C** head, lateral view **D** head, anterolateral view **E** head, anterior view. Scale bars: 1 mm.

#### Remarks.

**Figure 8.**
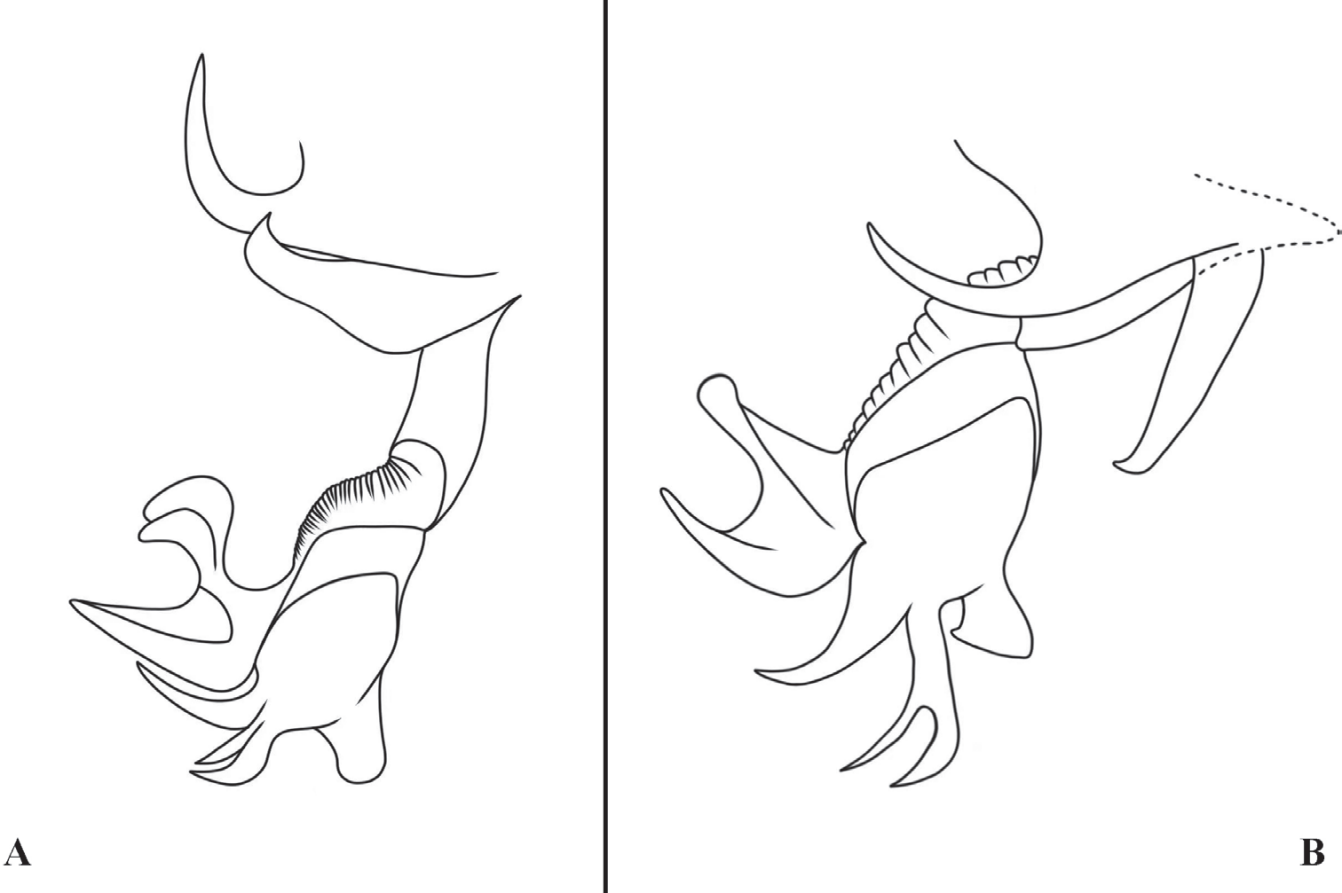
Sarcophaga (Phallantha) sichotealini Rohdendorf, 1938; phallus, lateral view **A** illustrated from figure 7B **B** adapted from Wei and Yang (2005, as *Pierretiadaozhenensis*).

The holotype of *P.daozhenensis* is deposited in CDCP and not currently available for loan and study. This nominal species was not included by Verves (2020), probably in an oversight. Wei (2005) described the vesica as flower-like, the cerci as having pointed apices and being slightly bent in lateral view, and the juxtal extension as well developed, flexed at its base and bent forward apically (Fig. 4). All of these features are consistent with *S.sichotealini*, and we consider the illustrations of the phallus provided by Xue and Chao (1998: 677, fig. 1332 m), and Wei and Yang (2005: fig. 3) to be a fully acceptable match (Fig. 8). We notice that Wei and Yang (2005) mentioned that *P.daozhenensis* was assigned to *Pierretia* using the key by Xue and Chao (1998), but the species was not assigned to any of the subgenera applied by Xue and Chao (1998), which includes *Phallantha*. Wei and Yang (2005) made no discussion about the subgeneric affiliation of *P.daozhenensis*, and there is no comparison with *P.sichotealini* in spite of the significant similarities with the illustration provided by Xue and Chao (1998). Vesica and harpes are of the same overall configuration, and as these are composed of flattened, partly membranous structures, even small changes in orientation may result in considerable changes in outline. The juxta has a very characteristic shape, with an almost exact match. Sarcophaga (P.) sichotealini is distributed in China (Guizhou, Hunan, Sichuan, Yunnan), the Russian Far East, South Korea, and temperate Japan (Pape 1996; Xue and Chao 1998; Verves 2020).

### ﻿Subgenus Pterosarcophaga

Taxon classificationAnimaliaDipteraSarcophagidae

Ye, 1981

92218CE2-A543-5AE3-99D4-8AD12DF2E7A9

Pterosarcophaga Ye, 1981: 229. Type species: Pterosarcophagaemeishanensis Ye & Ni, 1981, by original designation.
Pterosacophaga : Ye 1981: 230. Incorrect original spelling of Pterosarcophaga, by First Reviser action of Ye (1982: 21).
Magnicauda Wei, 2005: 405. Type species: Magnicaudalinjiangensis Wei, 2005, by original designation. Syn. nov.
Maginicauda : Wei 2005: 409. Incorrect original spelling of Magnicauda, by First Reviser action in the present paper.

#### Remarks.

Monotypic subgenera in *Sarcophaga* (*sensu lato*) are often erected for lack of evidence as to their phylogenetic relationships, and as such they convey little if any information. We prefer a classification based on similarities rather than on differences, and as Wei (2005) considered *Magnicauda* to be closely related to *Pterosarcophaga* due to the male cerci of the type species of both subgenera being expanded, wing-like, in lateral view, we are here treating the two nominal subgenera as synonyms.

Ye in Ye and Ni (1981) provided two different spellings: “*Pterosarcophaga*” (pp. 229, 232, 233) and “*Pterosacophaga*” (p. 230). By using only the spelling “*Pterosarcophaga*”, Ye (1982: 21) acted as First Reviser according to Article 24.2.4 of the Code.

Wei (2005) provided two different spellings: “*Magnicauda*” (pp. 404–406, 408) and “*Maginicauda*” (p. 409). Since then, the only mention of this genus-group name we have found is that of Verves (2020: 48); however, as only the spelling “*Maginicauda*” was used, the criteria of Article 24.2.3 for a First Reviser action were not fulfilled. Wei (2005) did not provide an explicit etymology, but the description of a remarkably broad male cercus is here taken to indicate that “*Magnicauda*” was the intended spelling. This is supported by the repeated use of this spelling, whereas the spelling “*Maginicauda*” was used only once. We herewith select “*Magnicauda*” to be the correct original spelling, by First Reviser action.

### ﻿ Sarcophaga (Pterosarcophaga) linjiangensis

Taxon classificationAnimaliaDipteraSarcophagidae

(Wei, 2005)

08E3DB5C-A3D5-50FD-978E-62A62C5AA855

Fig. 9

Magnicauda linjiangensis Wei, 2005: 405. China, Guizhou, Xishui, Linjiang National Nature Reserve.
*linjianensis*: Wei 2005: 408, incorrect original spelling of *linjiangensis* Wei, 2005, by First Reviser action in the present paper.

#### Material examined.

None.

#### Remarks.

This species can be distinguished from other species of *Sarcophaga* by the flag-like pregonite. Wei (2005) provided two different spellings: “*linjiangensis*” (pp. 404–406, 409) and “*linjianensis*” (p. 408). Since then, the only mention of the species we have found is that of Verves (2020: 48); however, as only the spelling “*linjiangensis*” was used, the criteria for a First Reviser action were not fulfilled (see Art. 24.2.3 of the Code). As the species was evidently named after its type locality, we herewith select “*linjiangensis*” as the correct original spelling by First Reviser action.

**Figure 9.**
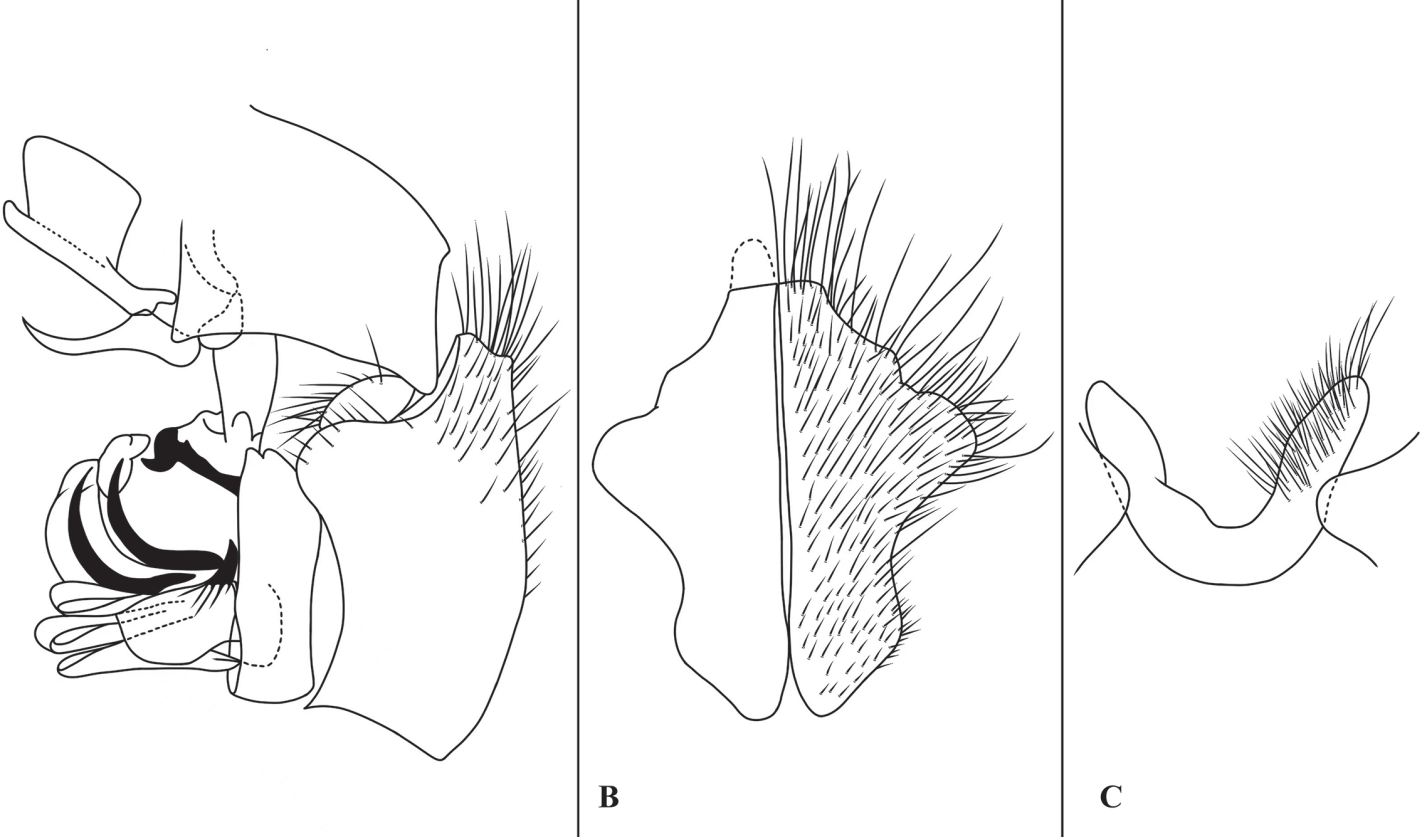
Sarcophaga (Pterosarcophaga) linjiangensis (Wei, 2005); male terminalia **A** terminalia, lateral view **B** cerci, dorsal view **C** sternite 5, ventral view. (Adapted from Wei 2005).

### ﻿Subgenus Robineauella

Taxon classificationAnimaliaDipteraSarcophagidae

Enderlein, 1928

B8866048-8C5D-5D52-8C64-27CAA5AEDA08

Robineauella Enderlein, 1928: 23 (as subgenus of Parasarcophaga Johnston & Tiegs, 1921). Type species: Sarcophagascoparia Pandellé, 1896 [= Sarcophagacaerulescens Zetterstedt, 1838], by original designation.

### ﻿ Sarcophaga (Robineauella) huangshanensis

Taxon classificationAnimaliaDipteraSarcophagidae

(Fan, 1964)

3E21F534-24B5-5EC2-A9E0-735420EEDD58

Figs 10 , 11 , 12

Parasarcophaga (Robineauella) huangshanensis Fan, 1964: 312. China, Anhui, Huang-Shan.
Parasarcophaga simultaneousa Wei & Yang, 2007: 528. China, Guizhou, Leigongshan. Syn. nov.

#### Material examined.

***Holotype*** of Parasarcophaga (Robineauella) huangshanensis Fan, 1964: ♂, China, Anhui, Huangshan, 19.vi.1936, [unknown collector] [terminalia not recovered]. ***Paratypes***: 2♂♂, China, Zhejiang, Tianmu mountain, 1100 m, 5.vii.1962, Zhizi Chen leg. (SECA).

**Figure 10.**
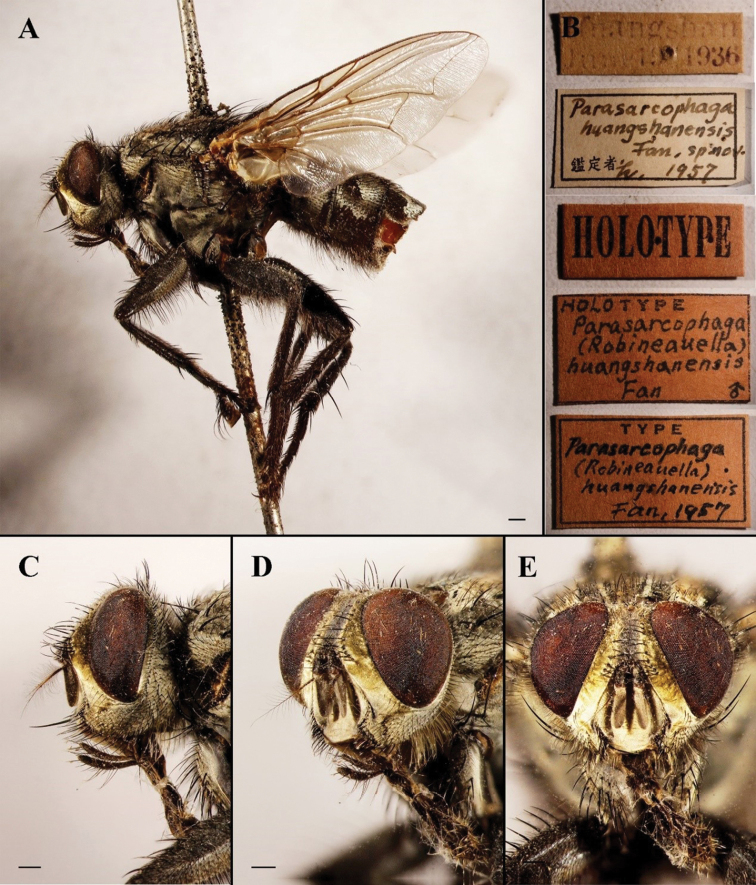
Sarcophaga (Robineauella) huangshanensis (Fan, 1964); holotype (China, Anhui; in SECA) **A** habitus, lateral view **B** labels **C** head, lateral view **D** head, anterolateral view **E** head, anterior view. Scale bars: 1 mm.

#### Remarks.

**Figure 11.**
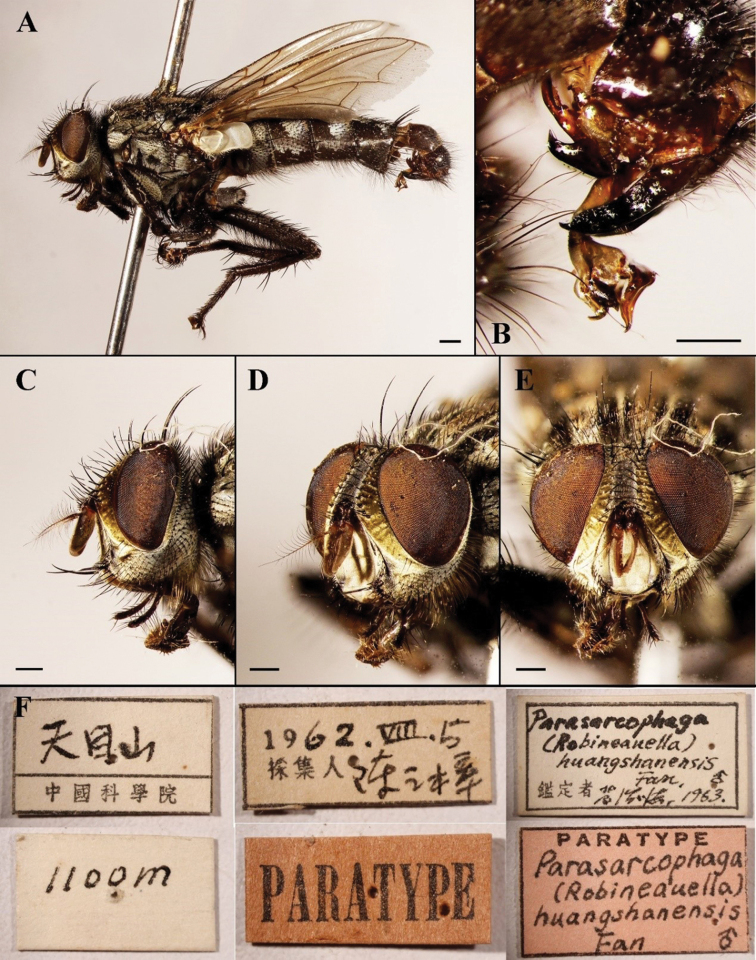
Sarcophaga (Robineauella) huangshanensis (Fan, 1964); paratype (China, Anhui; in SECA) **A** habitus, lateral view **B** terminalia, lateral view **C** head, lateral view **D** head, anterolateral view **E** head, anterior view **F** labels. Scale bars: 1 mm.

**Figure 12.**
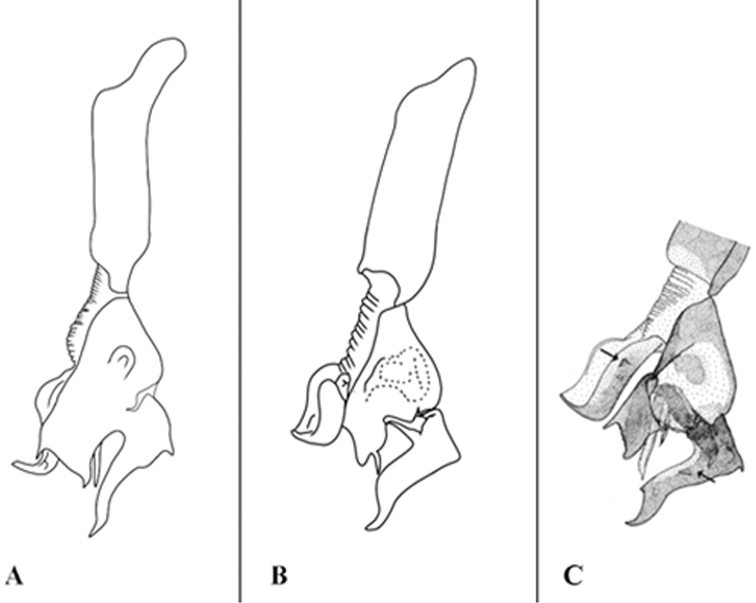
Sarcophaga (Robineauella) huangshanensis (Fan, 1964); phallus, lateral view **A** adapted from Fan (1964)**B** adapted from Wei and Yang (2007, as *Parasarcophagasimultaneousa*) **C** adapted from Lehrer (2012, as *Robineauellasimultaneousa*).

We examined the type series of S. (R.) huangshanensis and found that the male terminalia are a close match with the description and illustrations provided for *P.simultaneousa* (Figs 11, 12). The most important difference would be the difference in thickness of the proximal part of the juxtal processes, but this is here considered as infraspecific variation. Wei and Yang (2007) noted that this species could be confused with S. (Liosarcophaga) kitaharai Miyazaki, 1958; however, the latter, as a member of *Liosarcophaga* Rohdendorf, 1937, has a distiphallus with a better-developed dorso-median juxtal extension and an almost right-angled apico-dorsal part of juxta (Figs 11b, 12b, c). Lehrer (2012) examined the holotype of *S.simultaneousa* and mentioned a similarity to *R.daurica* Grunin, 1964 and *R.mendeliana* Lehrer, 2008 (as “*mendelliana*”); however, he did not mention S. (R.) huangshanensis, maybe by an oversight.

## Supplementary Material

XML Treatment for Sarcophaga

XML Treatment for Bellieriomima

XML Treatment for Sarcophaga (Bellieriomima) genuforceps

XML Treatment for Cornexcisia

XML Treatment for Sarcophaga (Cornexcisia) suthep

XML Treatment for Liosarcophaga

XML Treatment for Sarcophaga (Liosarcophaga) aegyptica

XML Treatment for Sarcophaga (Liosarcophaga) kanoi

XML Treatment for Phallantha

XML Treatment for Sarcophaga (Phallantha) sichotealini

XML Treatment for Pterosarcophaga

XML Treatment for Sarcophaga (Pterosarcophaga) linjiangensis

XML Treatment for Robineauella

XML Treatment for Sarcophaga (Robineauella) huangshanensis
